# Labour epidural analgesia and early childhood behavioural outcomes: the moderating role of maternal mental health and pro‐inflammatory cytokines

**DOI:** 10.1111/anae.70203

**Published:** 2026-03-15

**Authors:** Sana Asif, Hsing‐Fen Tu, Richard A. White, Birgitta Birgisdottir, Miklos Lipcsey, Martin F. Bjurström, Alkistis Skalkidou

**Affiliations:** ^1^ Department of Surgical Sciences Anaesthesiology and Intensive Care Medicine Uppsala University Uppsala Sweden; ^2^ Department of Women's and Children's Health Uppsala University Uppsala Sweden; ^3^ Department of Psychology Uppsala University Uppsala Sweden; ^4^ Department of Applied Educational Science Umeå University Umeå Sweden; ^5^ Hedenstierna Laboratory, Department of Surgical Sciences Uppsala University Uppsala Sweden; ^6^ Clinical Pain Research, Department of Surgical Sciences Uppsala University Uppsala Sweden

**Keywords:** CBCL scores, labour epidural analgesia, late gestation cytokines, postpartum depression, resilience

## Abstract

**Introduction:**

Labour epidural analgesia is the most effective method for intrapartum pain relief and is associated with improved maternal outcomes. However, concerns have been raised regarding potential associations between labour epidural analgesia and adverse psycho‐emotional outcomes in children. Evidence from large epidemiological studies is inconsistent and potential biological mechanisms remain unclear. Maternal immune activation during pregnancy may play a role. We aimed to investigate behavioural and psycho‐emotional outcomes in children of mothers who received epidural analgesia during labour, accounting for perinatal mental health, sociodemographic characteristics and cytokine profiles.

**Methods:**

Singleton vaginal births from the Biology, Affect, Stress, Imaging and Cognition study were included. Child behavioural outcomes were assessed by Child Behavioural Checklist scores at 18 months, 6 years and 11 years postpartum in the U‐BIRTH follow‐up cohort, with higher scores indicating more behavioural difficulties. The main exposure was maternal use of labour epidural analgesia. Maternal data were collected from questionnaires and medical records.

**Results:**

Among 1962 mother–child dyads, 726 (37%) received labour epidural analgesia. Younger maternal age; lower resilience; inflammatory diseases; primiparity; antenatal depression; fear of childbirth; and longer duration of labour were associated with higher Child Behavioural Checklist scores at 18 months postpartum. In crude analysis, labour epidural analgesia correlated with higher Child Behavioural Checklist scores at 18 months postpartum; however, this association was not significant after adjusting for confounders. Among those with lower expression of TNFSF14 and CXCL6 cytokines, labour epidural analgesia use was associated with higher Child Behavioural Checklist scores.

**Discussion:**

Use of epidural analgesia during labour was not found to be independently associated with adverse child behavioural outcomes. Variations in maternal cytokine profiles among those choosing labour epidural analgesia or not may influence susceptibility to early behavioural differences. Replication in larger cohorts and further exploration of additional immune biomarker dynamics during pregnancy are warranted.

## Plain Language Summary

**What we did:**

We studied almost 2000 mothers and their children to see if using an epidural for pain relief during labour was linked to children's behaviour later in life. We looked at children's behaviour when they were 18 months old, 6 years old and 11 years old using standard behaviour questionnaires. We also collected information about the mothers, such as their mental health, background and levels of certain immune system chemicals in their blood.

**Why did we do it:**

Epidurals are very good at reducing pain during childbirth, but some people worry they might affect a child's emotional or behaviour development. Past studies have given mixed results, and it is not clear why an epidural would cause any effect. We wanted to find out if epidurals were linked to children's behaviour after taking into account other important things, such as the mother's health stress, and immune system activity during pregnancy.

**What we found:**

At first, children whose mothers had an epidural seemed to have slightly more behaviour difficulties at 18 months. However, after we allowed for other factors like the mother's age, mental health, fear of childbirth and length of labour, the epidural itself was not linked to worse behaviour in children. Some children whose mothers had certain immune system patterns did show small differences in behaviour, but overall, epidurals were not found to cause behaviour problems in children. This suggests epidurals are not harmful to children's emotional or behaviour development, although more research is needed to understand how the immune system in pregnancy might play a role.

## Introduction

Epidural analgesia remains the most effective modality to alleviate pain during labour [1]. In addition to lower pain intensity, epidurals also increase satisfaction and decrease rates of maternal life‐threatening outcomes [1, 2]. Benefits associated with epidural analgesia have led to widespread use, not least in high‐income countries, where as many as four out of five first‐time mothers receive labour epidural analgesia [3]. Importantly, use of epidural analgesia during labour, compared with non‐epidural methods, does not appear to cause any significant adverse obstetric or early neonatal outcomes, although first and second stages of labour may be prolonged [1].

Despite substantial maternal health effects, concerns have been raised regarding an association between use of epidural analgesia during labour and impaired neurodevelopment in children. These concerns are based on a number of large, retrospective population‐based birth cohort studies which have shown significantly increased hazard ratios when evaluating the association between exposure to labour epidural analgesia and subsequent development of conditions such as autism spectrum disorders [4, 5, 6]. In parallel, several large epidemiological studies have found no associations between maternal epidural analgesia use and adverse neurodevelopmental and behavioural outcomes [7, 8, 9, 10]. Various factors may explain these heterogeneous findings, such as baseline differences in cohorts, confounders related to treatment and analytical limitations, and the interpretation of these data is complex [11].

Multiple well‐characterised genetic and environmental risk factors are linked to adverse neurodevelopmental outcomes, independent of labour epidural analgesia. Variables that shift the gene:environment balance in a negative direction include perinatal complications (e.g. asphyxia) and maternal factors such as higher age, obesity and autoimmune diseases [12]. Given that epidural analgesia is typically used for a very brief period of time in the final stage of pregnancy, rather than the first trimester when dynamics of brain development peak, it is unclear how limited labour epidural analgesia exposure might impact neurodevelopment from a mechanistic perspective [13]. Importantly, although local anaesthetics can cross the placenta, concentrations in neonatal blood sampled during labour epidural analgesia are reassuringly low [14, 15]. Nevertheless, it is hypothesised that maternal immune activation throughout pregnancy, even in the perinatal period, may disrupt brain development through neuroinflammatory processes [12]. Although a direct link between labour epidural analgesia and brain injury remains unsubstantiated at present, approximately 15–25% of patients with labour epidural analgesia develop ‘*epidural‐related maternal fever*’ (possibly due to sterile inflammation and altered thermoregulation) [12]. Recent meta‐analytic data suggest that intrapartum hyperthermia of any cause is associated with neonatal brain injury [16]. Previous studies have indicated that elevated levels of maternal and/or neonatal biomarkers of inflammation (e.g. pro‐inflammatory cytokines and C‐reactive protein) are associated with impaired neurodevelopment, although these findings are inconclusive [12].

In this large, prospective cohort study including maternal, perinatal and clinical data, we investigated whether use of labour epidural analgesia influences risk of adverse neurodevelopmental outcomes in children, adjusting for relevant confounders, and explored the possible moderating role of immune activation through assessment of comprehensive late‐pregnancy maternal cytokine profiles in a subset of participants. To avoid ambiguity in the primary outcome assessment, the Child Behaviour Checklist (CBCL), a robust parent‐reported questionnaire, was used longitudinally across three time‐points, spanning a period of 11 years.

## Methods

This nested case control study was conducted within the longitudinal population‐based Biology, Affect, Stress, Imaging and Cognition (BASIC) study (2010–2019, Uppsala, Sweden) and its extension, the U‐BIRTH study. The BASIC study investigates the psychological well‐being of women during and after pregnancy [17]. All Swedish‐speaking women aged ≥ 18 y undergoing a routine ultrasound were invited to participate. Women aged < 18 y with: insufficient Swedish language proficiency; protected identity (i.e. confidentiality marking indicating protected data due to safety reasons); blood‐borne infections or non‐viable pregnancy detected during routine ultrasound; elective caesarean section; or twin pregnancy, were not included. The ongoing U‐BIRTH study, initiated in 2012, is a follow‐up study of BASIC mothers and their children [18]. Ethical approval was granted by the Swedish Ethical Review authority.

Participants completed web‐based questionnaires at gestational weeks 17 and 32, and at 6 weeks, 8 weeks and 6 months postpartum. Medical information was retrieved from medical records. Between 2010 and 2019, BASIC recruited 6478 pregnant women [17]. Those who participated in BASIC were invited to the U‐BIRTH study when their child was approximately 15 months old [19]. In the U‐BIRTH study, women completed web‐based surveys at 18 months, 6 years and 11 years postpartum [18]. The ongoing U‐BIRTH cohort has recruited 2675 mother–child dyads. At the time of analysis, the cohort included 97.2% of participants at 6 years and over half at 11 years postpartum. This study included women with singleton vaginal deliveries between 2010 and 2019 and with available U‐BIRTH data at 18 months postpartum.

The main outcome measure was the Swedish CBCL score, previously shown to have good psychometric properties, at 18 months, 6 years and 11 years postpartum [19, 20]. Two age‐appropriate CBCL versions were used: one for children aged 1.5–5 y (99 items); and one for children aged 6–18 y (113 items). These assessed emotional and behavioural problems, yielding total scores as well as internalising and externalising subscales [21, 22]. Items were rated on a 3‐point scale (0 = not true, 1 = somewhat or sometimes true, 2 = very true or often true). For CBCL 1.5–5 y, the internalising domain includes ‘Emotionally Reactive’; ‘Anxious/Depressed’; ‘Somatic Complaints’; and ‘Withdrawn’, while the externalising domain includes ‘Attention Problems’; and ‘Aggressive Behaviour’. For CBCL 6–18 y, the internalising domain includes ‘Anxious/Depressed’; ‘Withdrawn/Depressed’; and ‘Somatic Complaints’, while the externalising domain includes ‘Rule‐Breaking Behaviour’; and ‘Aggressive Behaviour’.

Three groups of variables were included in the analyses. The first group included social; resilience‐related; and medical and obstetric covariates. At gestational week 17, participants reported age; BMI; ethnicity (Caucasian vs. non‐Caucasian); education (> 16 vs. ≤ 16 years); employment status (working vs. unemployed/sick leave); intimate partner violence; cohabitation with child's father; smoking history; and resilience (high vs. low). Resilience was measured using the Swedish version of the 29‐item Sense of Coherence scale and low resilience was defined as a score < 128, corresponding to one standard deviation below the mean in the BASIC dataset [23]. Pre‐existing psychosomatic conditions retrieved from medical records included: history of depression; chronic pain disorders; chronic sleep problems; endocrine disorders; premenstrual syndrome (as per American College of Obstetricians and Gynaecologists criteria); inflammatory disorders (type 1 diabetes mellitus and/or irritable bowel syndrome and/or migraine); iron deficiency anaemia; family history of delayed speech; and use of analgesics, corticosteroids, thyroid hormone replacement and hormonal contraceptives within one year before or during pregnancy. Participants also reported on parity (multiparous vs. nulliparous) and assisted reproduction techniques.

The second group of variables included pregnancy and delivery covariates. At gestational week 32, participants reported fear of childbirth (any vs. none) and antenatal sleep duration (> 6 h vs. ≤ 6 h). Antenatal depression was assessed using the Edinburgh Postnatal Depression Scale and/or the Depression Self‐Rating Scale and/or the Mini International Neuropsychiatric Interview [24, 25, 26]. Antenatal depression was defined as Edinburgh Postnatal Depression Scale score ≥ 12 and/or a depressive episode according to Depression Self‐Rating Scale and/or depression episode according to the Mini International Neuropsychiatric Interview.

Information was gathered on pregnancy complications (any vs. none), either self‐reported or extracted from medical records. Delivery‐related information retrieved from medical records included: duration of pregnancy (≥ 287 vs. < 287 days); induction; oxytocin administration; duration of active and second stage of labour; mode of delivery (vaginal vs. vacuum extraction or emergency caesarean section); Apgar score < 7 at 5 min; premature birth; and child sex.

The remaining group of variables included the following postpartum covariates: delivery experience as reported at 6 weeks (positive vs. negative); perineal laceration problems; breastfeeding success; postpartum depression (defined as Edinburgh Postnatal Depression Scale score ≥ 12 and/or a depressive episode according to Depression Self‐rating Scale and/or a depression episode after the Mini International Neuropsychiatric Interview at 6 or 8 weeks postpartum and/or at 6 months postpartum); and mother–child bonding (assessed at 6 months postpartum using the 25‐item postpartum bonding questionnaire, with higher scores indicating greater bonding difficulties) [27].

Finally, we investigated whether maternal cytokine levels at gestational week 38 moderated any associations found between labour epidural analgesia and CBCL scores. Venous blood was collected at gestational week 38 and cytokine expression was analysed using Proseek Multiplex Inflammation I panel (Olink Bioscience, Uppsala, Sweden), based on proximity extension assay technology covering a total of 92 immune‐related cytokines [28, 29]. Normalised protein expression (log2 scale) was used for relative quantification. Cytokines with data available for > 60 participants were included resulting in a total of 26 cytokines with available data. Cytokines levels were characterised as high or low, with high defined as values ≥ 50th percentile and low defined as values < 50th percentile.

Group differences (labour epidural analgesia vs. non‐labour epidural analgesia) were calculated by χ^2^ or Mann–Whitney U tests or ANOVA when appropriate. Analyses were performed using SPSS (version 28.0; IBM SPSS, Armonk, NY, USA). Missing data were addressed using multiple imputation with chained equations. Twenty imputed datasets were generated separately for the full dataset and for the subset restricted to participants with cytokine data. Imputation models included all variables used in the respective analyses. Imputation was performed using the MICE package in R (R Foundation for Statistical Computing, Vienna, Austria), with default settings, and results were pooled across datasets by combining draws from the posterior distributions [30]. To estimate the association between peripartum labour epidural analgesia use and child behavioural outcomes, we fitted three sets of linear models for each CBCL outcome (externalising, internalising and total scores) at 18 months, 6 years and 11 years. The first set of models included only the exposure variable (use of labour epidural analgesia). The second set were penalised models that adjusted for all potential confounders using a hierarchical shrinkage prior. The third set were non‐penalised adjusted models that included only covariates with a p value equivalent < 0.05 in the penalised models. These covariates were: age; lower resilience; inflammatory disorders; fear of childbirth; duration of active labour phase; and duration of second stage of labour. All models were estimated using the rstanarm package in R with four Markov chains and 10,000 iterations. Posterior medians and 95% credible intervals were reported. A Bayesian approach was chosen so that uncertainty estimates could be obtained for the penalised regression.

To assess potential effect modification by inflammatory markers, cytokine levels were binarised at the median. This analysis was limited to the 61 women with available cytokine data. For the outcome of total CBCL score at 18 months postpartum, we fitted a series of Bayesian linear models, each including an interaction term between use of labour epidural analgesia and one binarised cytokine. Adjusted covariates included BMI, inflammatory disorders and gestational age at sampling, using few covariates due to limited sample size. Estimates were pooled and summarised using the same approach as in the main effects models. Due to the lack of a penalised model, a frequentist approach was chosen, and estimates were pooled and summarised using Rubin's rules [31].

All analyses were conducted using R version 4.3.1. Project organisation was managed using the org package. Imputation was performed with mice and Bayesian modelling with rstanarm [30, 31, 32].

## Results

**Figure 1:**
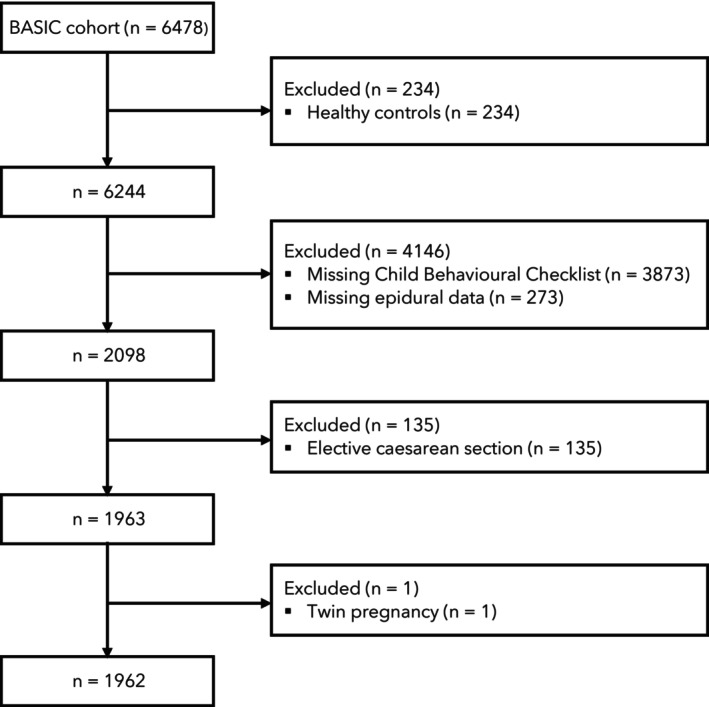
Study flow chart. BASIC, Biology, Affect, Stress, Imaging and Cognition (BASIC).

Crude descriptive characteristics by use of labour epidural analgesia were examined among 1962 mother–child dyads (Fig. 1), of whom 726 (37%) received labour epidural analgesia. This group was: younger; had higher BMIs; higher rates of intimate partner violence; and lower resilience scores. Compared with the non‐labour epidural analgesia group, this group was more likely to have a history of depression; endocrine or inflammatory disorders; premenstrual syndrome; and family history of delayed speech (Table 1). Labour epidural analgesia was common among primiparas, with pregnancy duration of > 287 days and in those reporting fear of childbirth or antenatal depression. Labour interventions were more frequent in labour epidural analgesia users.

**Table 1:** Maternal sociodemographic, medical, obstetric and postpartum characteristics (n = 1962) by use of labour epidural analgesia. Values are number and proportion of the total population or median (IQR [range]) stratified by labour epidural analgesia use.

Variables	No labour epidural analgesia		Labour epidural analgesia	
	1236/1962	63%	726/1962	37%
Age; y	1236/1236	32 (6 [18–45])	726/726	31 (6 [20–45])
BMI; kg.m^‐2^	1150/1236	22 (4 [17–39])	688/726	23 (5 [16–43])
Non‐Caucasian	25/850	2.9%	18/446	4.0%
Education < 16 years	179/1154	15.5%	118/687	17.2%
Working (full or part time)	1055/1152	91.6%	647/688	94.0%
Intimate partner violence	88/1120	7.9%	87/678	12.8%
Lower resilience	76/1236	6.1%	62/726	8.5%
Not cohabiting with child's father	68/1119	6.1%	28/651	4.3%
Smoking (ever)	277/1149	24.1%	222/691	32.1%
Existing psychosomatic comorbidities				
History of depression	580/1151	50.4%	396/689	57.5%
Chronic pain disorders	50/1236	4.0%	39/726	5.4%
Chronic sleep problems	35/1142	3.1%	22/691	3.2%
Endocrine disorders	30/1236	2.4%	30/726	4.1%
Premenstrual syndrome	106/848	12.5%	93/548	17.0%
Inflammatory disorders	538/1234	43.6%	357/724	49.3%
Iron deficiency anaemia	26/1236	2.1%	26/726	3.6%
Family history of delayed speech	165/1216	13.6%	77/722	10.7%
Medications before/during pregnancy				
Analgesics	116/1236	9.4%	101/726	13.9%
Corticosteroids	44/1236	3.6%	32/726	4.4%
Thyroid hormone replacement	80/1236	6.5%	46/726	6.3%
Hormonal contraceptives	353/1236	28.6%	261/726	36.0%
Obstetric characteristics				
Primiparous	457/1236	37.0%	499/726	68.7%
Assisted reproduction techniques	111/1234	9.0%	83/722	11.5%
Pregnancy complications	587/1074	54.7%	372/637	58.4%
Fear of childbirth	201/1168	17.2%	198/691	28.7%
Antenatal sleep duration	93/1167	8.0%	61/689	8.9%
Antenatal depression	187/1223	15.3%	136/723	18.8%
Pregnancy length ≥ 287 days	270/1236	21.8%	233/726	32.1%
Delivery characteristics				
Induced labour	185/1229	15.1%	221/720	30.7%
Oxytocin administration	144/523	27.5%	175/234	74.8%
Active phase of labour; h	442/1236	4 (5 [1–30])	363/726	10 (8 [1–30])
Second stage of labour; h	916/1236	0 (0 [0–4])	520/726	0 (0 [0–8])
Mode of delivery				
Vaginal	1111/1236	89.9%	493/726	67.9%
Vacuum extraction	47/1236	3.8%	119/726	16.4%
Emergency caesarean section	78/1236	6.3%	114/726	15.7%
Child characteristics				
Apgar score < 7 at 5 min	24/1166	2.1%	26/684	3.8%
Premature birth	65/1229	5.3%	18/720	2.5%
Sex; male	628/1236	50.8%	394/726	54.3%
Postpartum characteristics				
Negative delivery experience	39/1218	3.2%	85/709	12.0%
Perineal laceration problems	124/1168	10.6%	115/686	16.8%
Depression	228/1207	18.9%	184/707	26.0%
Unsuccessful breastfeeding	153/1140	30.3%	152/359	42.3%
Bonding difficulties	14/1072	1.3%	16/628	2.5%
Postpartum Child Behavioural Checklist scores				
18 months				
Total score	1236/1236	11 (12 [1–82])	726/726	13 (12 [1–60])
Externalising scores	1236/1236	6 (8 [0–32])	726/726	8 (8 [0–32])
Internalising scores	1236/1236	2 (4 [0–42])	726/726	3 (4 [0–29])
6 years				
Total score	520/1236	10 (13 [0–199])	315/726	11 (13 [0–71])
Externalising scores	565/1236	(6 [0–66])	338/726	4 (6 [0–32])
Internalising scores	582/1236	2 (4 [0–64])	348/726	3 (5 [0–27])
11 years				
Total score	264/1236	9 (15 [0–74])	129/726	10 (14 [0–197])
Externalising scores	289/1236	2 (4 [0–30])	142/726	3 (6 [0–70])
Internalising scores	290/1236	4 (7 [0–33])	140/726	3 (6 [0–61])

CBCL scores assessed by CBCL version 1.5–5 at 18 months and CBCL version 6–18 years at 6 years and 11 years postpartum.

Crude descriptive characteristics and CBCL scores were examined in relation to maternal and perinatal factors. Higher CBCL scores at 18 months postpartum were associated with: younger age; education < 16 years; intimate partner violence; inflammatory disorders; primiparity; fear of childbirth; antenatal depression; prolonged labour; postpartum depression; unsuccessful breastfeeding; and bonding difficulties (Table 2). Child Behavioural Checklist scores across all domains at 18 months postpartum were correlated with scores at 6 years and 11 years.

**Table 2:** Maternal sociodemographic, medical, obstetric and postpartum characteristics (n = 1962), correlated to Child Behaviour Checklist (CBCL) score at 18 months postpartum. Values are number/total population, median (IQR [range]) or Spearman's correlation coefficient, according to CBCL total, externalising and internalising scores at 18 months postpartum.

	Number	CBCL (total)	p value	CBCL (externalising)	p value	CBCL (internalising)	p value
Age; y	1962/1962	‐0.163	< 0.001	‐0.133	< 0.001	‐0.165	< 0.001
BMI; kg.m^‐2^	1838/1962	0.016	0.493	0.023	0.327	0.011	0.642
Non‐Caucasian	43/1296	13 (15 [1–40])	0.591	7 (9 [0–21])	0.742	2 (6 [0–14])	0.459
Education (< 16 years)	297/1841	13 (15 [1–60])	0.008	7 (10 [0–28])	0.043	3 (5 [0–29])	< 0.001
Working (full or part time)	1702/1840	12 (13 [1–60])	0.868	7 (8 [0–32])	0.966	3 (4 [0–29])	0.459
Intimate partner violence	175/1798	12 (13 [0–60])	0.152	7 (10 [0–30])	0.386	3 (4 [0–29])	0.036
Lower resilience	138/1962	16 (16 [2–51])	< 0.001	9 (10 [0–32])	< 0.001	3 (6 [0–26])	< 0.001
Not cohabiting with child's father	96/1770	11 (12 [1–41])	0.313	5 (7 [0–26])	0.269	2 (4 [0–15])	0.401
Smoking (ever)	499/1840	13 (13 [1–60])	0.017	7 (8 [0–32])	0.074	3 (5 [0–29])	0.011
Existing psychosomatic comorbidities							
History of depression	976/1840	12 (13 [1–82])	0.002	7 (9 [0–32])	0.050	3 (4 [0–42])	< 0.001
Chronic pain disorders	89/1962	13 (15 [1–82])	0.040	7 (9 [0–32])	0.146	3 (6 [0–42])	0.005
Chronic sleep problems	57/1833	13 (17 [1–53])	0.117	7 (8 [0–32])	0.490	4 (6 [0–26])	0.028
Endocrine disorders	60/1962	12 (11 [2–53])	0.915	6 (7 [0–24])	0.486	2 (4 [0–20])	0.879
Premenstrual syndrome	199/1396	13 (14 [1–54])	0.005	8 (9 [0–26])	0.010	3 (5 [0–26])	0.060
Inflammatory disorders	895/1958	13 (12 [1–82])	< 0.001	7 (8 [0–32])	< 0.001	3 (4 [0–42])	< 0.001
Iron deficiency anaemia	52/1962	14 (18 [2–41])	0.082	8 (9 [0–21])	0.062	3 (7 [0–14])	0.135
Family history of delayed speech	242/1938	12 (14 [1–82])	0.445	7 (8 [0–32])	0.540	3 (4 [0–42])	0.165
Medications before/during pregnancy							
Analgesics	217/1962	13 (11 [1–82])	0.094	7 (8 [0–32])	0.305	3 (5 [0–42])	0.029
Corticosteroids	76/1962	13 (16 [1–51])	0.025	7 (9 [0–32])	0.075	3 (6 [0–16])	0.086
Thyroid hormone replacement	126/1962	13 (11 [2–53])	0.026	7 (7 [0–30])	0.073	3 (4 [0–20])	0.034
Hormonal contraceptives	614/1962	12 (12 [1–60])	0.031	7 (8 [0–30])	0.096	3 (4 [0–29])	0.055
Obstetric characteristics							
Primiparous	956/1962	13 (12 [1–60])	< 0.001	7 (7 [0–32])	< 0.001	3 (4 [0–29])	< 0.001
Assisted reproduction techniques	194/1956	12 (13 [1–53])	0.088	7 (8 [0–26])	0.301	3 (5 [0–20])	0.032
Pregnancy complications	959/1711	12 (13 [1–60])	0.038	7 (8 [0–32])	0.085	3 (4 [0–29])	0.091
Fear of childbirth	399/1859	13 (14 [1–60])	< 0.001	8 (9 [0–32])	< 0.001	3 (5 [0–29])	< 0.001
Antenatal sleep duration	154/1856	13 (14 [1–51])	0.087	7 (8 [0–32])	0.236	3 (5 [0–26])	0.139
Antenatal depression	323/1946	14 (13 [1–54])	< 0.001	8 (9 [0–32])	0.001	3 (4 [0–26])	< 0.001
Pregnancy length ≥ 287 days	503/1962	13 (11 [1–82])	0.228	7 (8 [0–32])	0.756	3 (4 [0–42])	0.134
Delivery characteristics							
Induced labour	406/1949	13 (13 [1–60])	0.078	7 (8 [0–32])	0.352	3 (4 [0–29])	0.025
Oxytocin administration	319/757	12 (12 [1–82])	0.014	7 (7 [0–32])	0.160	3 (4 [0–42])	0.005
Active phase of labour; h	805/1962	0.102	0.004	0.084	0.017	0.100	0.005
Second stage of labour; h	1436/1962	0.109	< 0.001	0.089	< 0.001	0.116	< 0.001
Mode of delivery							
Vaginal	1604/1962	12 (13 [1–82])	0.050	6 (8 [0–32])	0.215	2 (4 [0–42])	0.118
Vacuum extraction	166/1962	13 (12 [1–53])		7 (7 [0–30])		3 (5 [0–20])	
Emergency caesarean section	192/1962	13 (11 [1–60])		7 (7 [0–27])		3 (4 [0–29])	
Child characteristics							
Apgar score < 7 at 5 min	50/1850	12 (9 [2–40])	0.997	7 (7 [0–21])	0.967	2 (3 [0–14])	0.755
Premature birth	83/1949	9 (10 [1–38])	0.043	5 (8 [0–21])	0.084	2 (4 [0–13])	0.274
Sex; male	1022/1962	13 (12 [1–82])	0.108	7 (8 [0–32])	0.029	3 (4 [0–42])	0.264
Postpartum characteristics							
Negative delivery experience	124/1927	14 (13 [1–60])	0.012	7 (9 [0–30])	0.096	3 (5 [0–29])	0.003
Perineal laceration problems	239/1854	13 (12 [1–53])	0.066	7 (8 [0–30])	0.113	3 (4 [0–26])	0.016
Depression	412/1914	14 (13 [1–82])	< 0.001	8 (10 [0–32])	< 0.001	3 (4 [0–42])	< 0.001
Unsuccessful breastfeeding	305/864	14 (14 [1–51])	< 0.001	9 (9 [0–32])	< 0.001	3 (5 [0–26])	0.004
Bonding difficulties	30/1700	24 (16 [6–51])	< 0.001	15 (8 [5–29])	< 0.001	6 (5 [0–26])	< 0.001
Postpartum Child Behavioural Checklist scores							
6 years							
Total score	835/1962	0.429	< 0.001	0.408	< 0.001	0.338	< 0.001
Externalising scores	903/1962	0.355	< 0.001	0.372	< 0.001	0.247	< 0.001
Internalising scores	930/1962	0.287	< 0.001	0.227	< 0.001	0.296	< 0.001
11 years							
Total score	393/1962	0.396	< 0.001	0.354	< 0.001	0.368	< 0.001
Externalising scores	431/1962	0.310	< 0.001	0.299	< 0.001	0.282	< 0.001
Internalising scores	430/1962	0.302	< 0.001	0.277	< 0.001	0.351	< 0.001

CBCL scores assessed by CBCL version 1.5–5 at 18 months and CBCL version 6–18 years, at 6 and 11 years postpartum.

**Figure 2:**
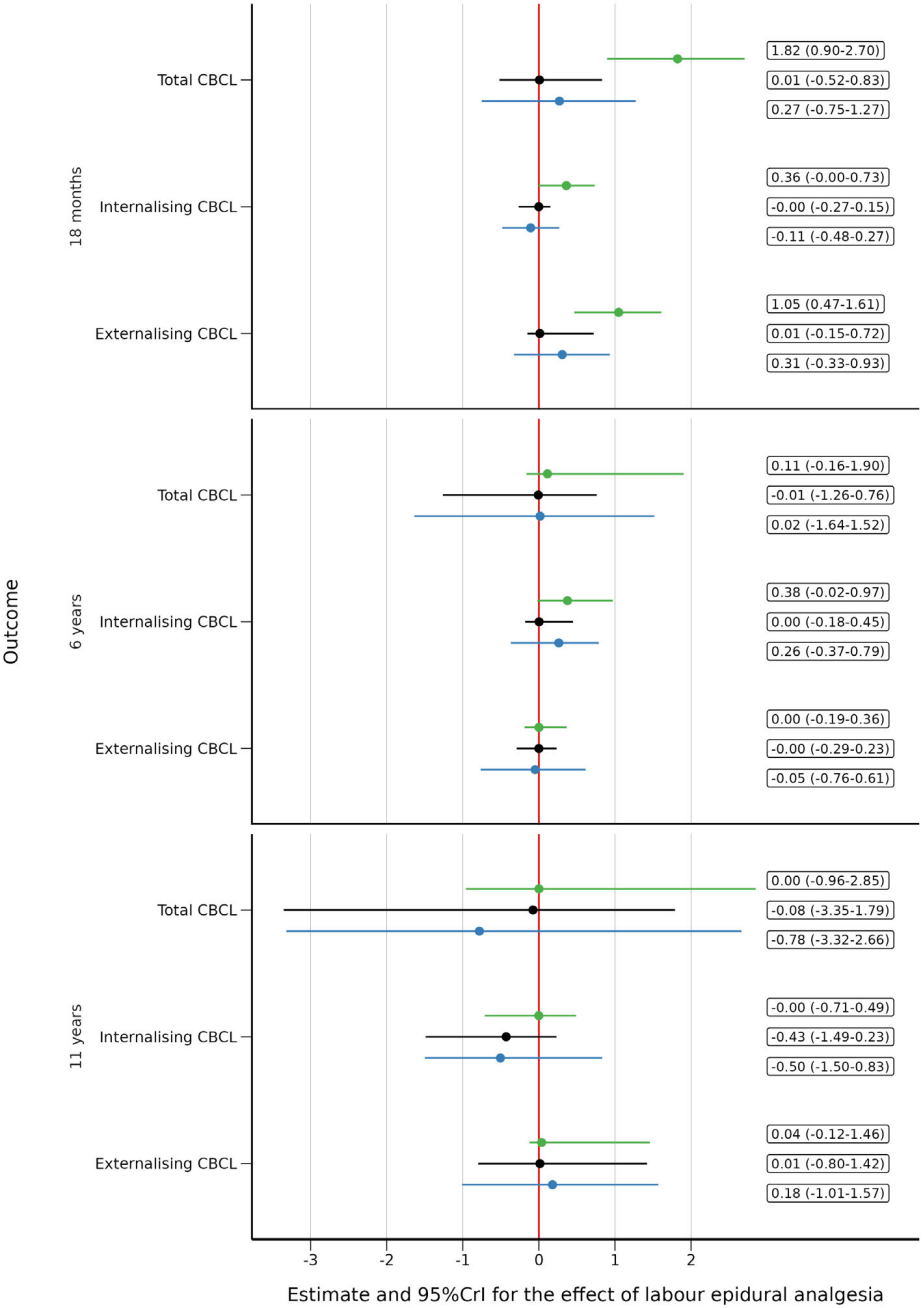
Association between labour epidural analgesia and Child Behaviour Checklist (CBCL) scores (total, externalising and internalising) at 18 months, 6 years and 11 years estimated β coefficients by regression models with 95% credible intervals (CrI). Green, crude; black, penalised; and blue, adjusted.

Association between maternal labour epidural analgesia use and CBCL scores was examined across early childhood. In the main‐effects analysis, use of labour epidural analgesia was associated with significantly higher CBCL total and externalising scores at 18 months postpartum in the crude model (Fig. 2). However, both adjusted and penalised estimates were non‐significant. No significant associations (crude, adjusted, penalised) were observed for any scores at later time‐points.

**Figure 3:**
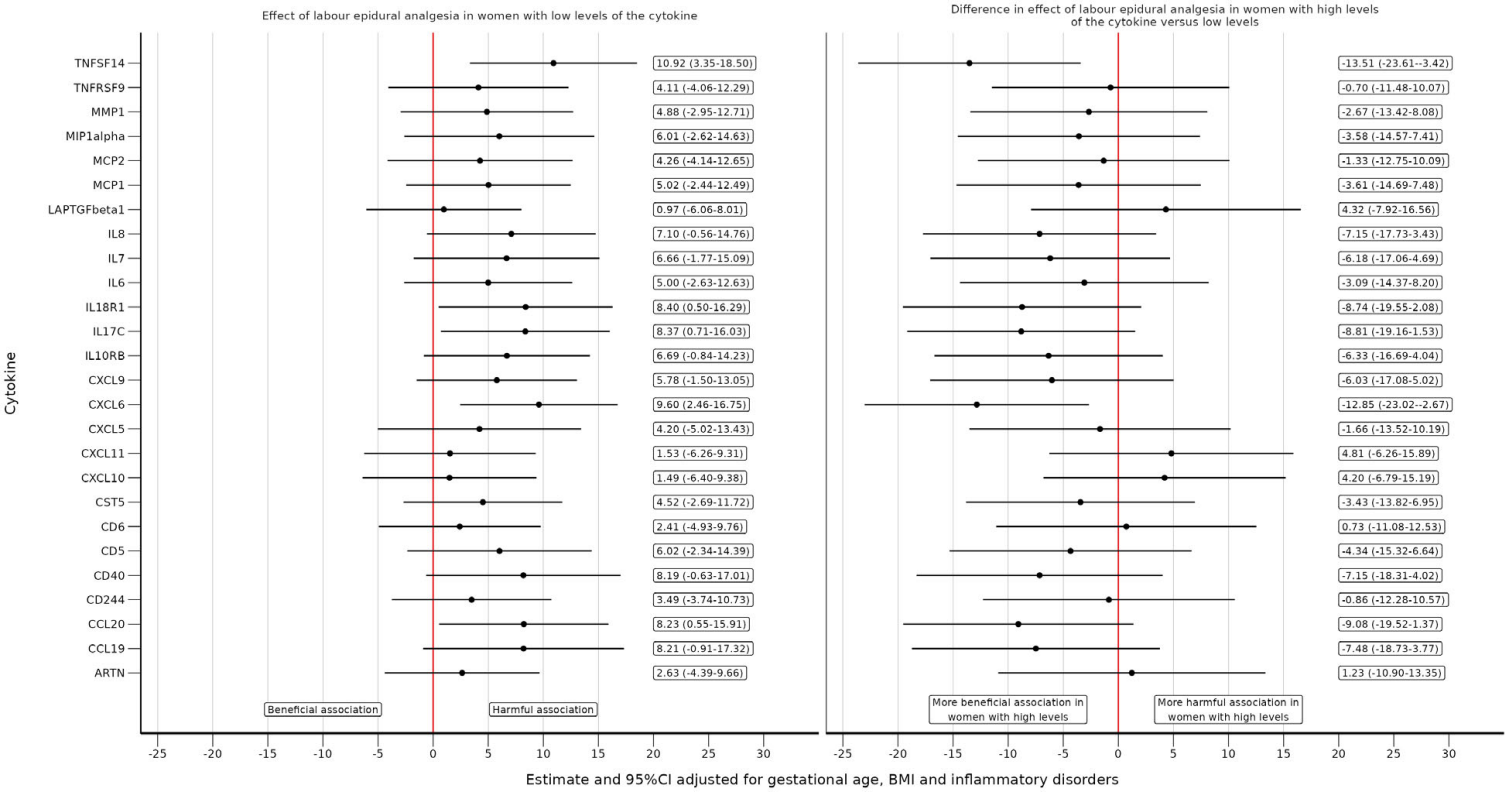
Between‐effect of labour epidural analgesia and cytokine expression at 38 weeks gestation (n = 61) in relation to Child Behaviour Checklist scores at 18 months postpartum. Low cytokine level was defined as < 50th percentile of cytokine expression, high cytokine level is defined as ≥ 50th percentile. Results are presented as estimated β coefficients and 95%CI.

Interaction analysis with maternal cytokines examined whether cytokine levels modified the association between maternal labour epidural analgesia and CBCL scores. Two cytokines – TNFSF14 and CXCL6 – modified the association between labour epidural analgesia and total CBCL scores at 18 months postpartum significantly. Among women with low levels of these cytokines in late pregnancy, use of labour epidural analgesia was associated with higher CBCL scores at 18 months, even in the adjusted models. In contrast, no significant association was observed among women with high cytokine levels (Fig. 3).

## Discussion

In this large longitudinal cohort of 1962 mother–child dyads, maternal labour epidural analgesia was not independently associated with adverse behavioural outcomes at 18 months, 6 years or 11 years postpartum, after adjusting for maternal psychological, obstetric and immunological factors. Exploratory analyses in a subset of 61 women suggested that maternal cytokine profiles may influence child behavioural vulnerability.

While early studies suggested a possible correlation between labour epidural analgesia and autism spectrum disorder, subsequent large‐scale and sibling‐matched studies found no consistent association [5, 7, 33]. Similarly, evidence for other developmental outcomes is mixed, with some studies reporting transient developmental delays while others observed no adverse effects [11, 33].

In our cohort, children of mothers who had labour epidural analgesia had higher CBCL total and externalising scores, but these associations disappeared after adjusting for confounders. No associations, in either crude or adjusted analyses, were observed at later time‐points, suggesting that early postnatal CBCL variations may reflect transient developmental or environmental influences rather than casual effects of labour epidural analgesia. Nevertheless, other factors not included in our analyses, such as paternal influences, social support or broader environmental conditions, may also play a role in early child behavioural outcomes and should be considered in future research [34].

Our exploratory analyses suggest that TNFSF14 and CXCL6 cytokines moderate the association between perinatal labour epidural analgesia use and child neurodevelopment (CBCL scores) at 18 months postpartum. TNFSF14, a tumour necrosis factor superfamily member, regulates inflammation and apoptosis [35]. At the same time, it has been shown that CXCL6 affects blood–brain barrier permeability and neurogenesis [36]. Dysregulation of these cytokines has been linked to various neuropsychiatric conditions [37, 38]. Although labour epidural analgesia can trigger sterile inflammation, hyperthermia occurs in only 15–20% of exposed women, suggesting substantial heterogeneity in maternal immunogenetic responses [12, 39]. Independent of labour epidural analgesia, maternal systemic inflammation due to infections, autoimmunity or high BMI has been shown to be associated with increased neurodevelopmental risks [12]. Disruptions in normal maternal immune response may increase susceptibility to adverse neurodevelopmental outcomes in children [12]. In our study, labour epidural analgesia use was associated with higher CBCL scores only among women with low cytokine levels, even after adjusting for confounders. This suggests that the maternal immune response to labour epidural analgesia may modify vulnerability to neurodevelopmental effects in children. However, given the limited sample size, these findings are preliminary and should be interpreted with caution.

A key strength of this study is that it is among the first to integrate maternal immune profiles with behavioural assessments. Associations between maternal labour epidural analgesia use and repeated CBCL scores were analysed longitudinally over 11 years, accounting for a comprehensive set of sociodemographic, resilience‐related, medical, psychiatric and obstetric factors. Multivariate models incorporated broad and targeted confounders, ensuring robust and reliable findings. Other strengths include prospective collection of self‐reported depressive symptoms, linkage to medical records and inclusion of biological, obstetric and psychological covariates, minimising recall bias. The longitudinal design and large sample size enhance reliability. The CBCL is a well‐validated tool, which reliably distinguishes children with and without behavioural and emotional problems; predicts later Diagnostic and Statistical Manual of Mental Disorders (DSM‐IV) diagnoses with good accuracy; discriminates between diverse neuropsychiatric disorders; and measures psychopathology and competence. This makes it a comprehensive tool for child mental health assessment [19].

Limitations include the low participation rate for the BASIC and U‐BIRTH studies, which may negatively impact generalisability. Participants were aware that the study focused on perinatal mental health, potentially introducing selection bias. In addition, highly educated mothers were over‐represented, whereas few mothers born outside of Sweden were included; the fact that questionnaires were only provided in Swedish may limit generalisability further. The rate of labour epidural analgesia use was 37%, which is lower than levels reported internationally but consistent with national estimates [40]. A further limitation is the small sample size related to cytokine analyses, which limits statistical power. Additional studies in larger cohorts are needed to confirm these results.

In conclusion, the current study found no evidence that exposure to labour epidural analgesia is associated with adverse child behavioural outcomes. Early CBCL differences at 18 months postpartum were not detectable after adjustment and were not seen at later ages, suggesting possible transient influences rather than causal effects of labour epidural analgesia. Exploratory analyses further indicated that maternal cytokine profiles may influence susceptibility, highlighting a potential role of prenatal immune variation in early child development. Replication of these findings in larger cohorts and further investigation of additional immune biomarkers during pregnancy are warranted to clarify these observations. Our findings indicate that labour epidural analgesia is unlikely to have long‐term adverse effects on child behavioural development.
